# Acoustic monitoring with miniature drones shows reduced Myotis bat occurrence with altitude and drone movement

**DOI:** 10.1038/s41598-025-96255-5

**Published:** 2025-04-10

**Authors:** Lauren Dobie, David M. Bird, Kyle H. Elliott

**Affiliations:** Department of Natural Resource Sciences, McGill University, Sainte-Anne-de-Bellevue, Quebec, H9X 2E3 Canada

**Keywords:** Aeroecology, Bats, Drones, Acoustic survey, Wildlife monitoring, Ecology, Behavioural ecology, Conservation biology

## Abstract

Our understanding of aeroecology is hampered by the challenge of sampling the air column, especially for nocturnal species like bats that forage high in the airspace. Nonetheless, monitoring endangered bat populations is vital for conservation efforts. Drones (unoccupied aerial vehicles) offer a relatively safe, cost-effective, and non-intrusive option for studying aerial wildlife. Here, we present a method for measuring bat distribution in the airspace using miniature drones (Mavic Mini 3 Pro) that are small enough to have negligible impacts on the bats themselves. We investigate how habitat, drone altitude, and drone movement influence bat sampling efficiency. A hovering drone detected more bats per minute than a moving drone for the EPNO complex (*Eptesicus fuscus* and *Lasionycteris noctivagans*) and all bats. Thus, we recommend the use of hovering point counts for surveying bats via drone. The *Myotis* complex (*M. septentrionalis*, *M. lucifugus*, and *M. leibii*) was more frequently present at lower altitudes over the sampled range (0–60 m). We conclude that bat taxa differentially occupy sectors of the air column, and that bat density in the airspace can be efficiently monitored by miniature drones.

**Supplementary Information:**

The online version contains supplementary material available at 10.1038/s41598-025-96255-5.

## Introduction

Drones are an emerging technology in ecological research, offering a relatively safe, cost-effective, and non-intrusive option for studying wildlife^1,2^. They have proven to be more accurate than traditional surveys in various ecological studies (seabirds^3^, marine wildlife^4^, and deer^5^). Drones have also introduced a new method for studying cryptic aerial species, such as bats, which occupy the airspace at night when individuals outside the detection range of traditional acoustic recorders may be difficult to observe^6–9^. Nonetheless, drones can impact wildlife by causing evasive behaviour^10,11^ or physiological responses, even in the absence of altered behaviour^12^.

Bat populations in Quebec are rapidly declining, with seven of eight species listed as Endangered, and we urgently need better ways of monitoring their abundance^13–16^. Since the year 2000, millions of bats have died in North America from white-nose syndrome, resulting in local extinctions of all hibernating bat species on the continent and causing significant conservation challenges^17^. Since bats are nocturnal, cryptic and aerial, sampling them can be challenging. Various methods are used to sample bats, such as mist nets, observational counts, and acoustic surveys. Acoustic surveys, conducted using autonomous recording units (ARUs) or hand-held bat detectors, are most useful when studying high-intensity echolocators^18^, such as the bat species in Quebec, and they often detect more species than with capturing techniques^19^. Using drones to conduct acoustic surveys could be beneficial by reducing sampling time and accessing remote areas and higher altitudes.

Although drones can be excellent tools for ecological research, their presence also raises concerns about potential negative interactions with wildlife, which should be considered when planning research involving drones. Few studies have used drones to conduct acoustic surveys of bats, and those that have used larger drones^6–9,20,21^. These larger drones caused reduced bat abundance due to increased noise and the presence of a large obstacle in the sky^6,22^. However, Kuhlmann et al. found that smaller, quieter drones have a lesser impact on acoustic bat surveys than larger, louder ones^22^. In particular, the DJI Mavic Mini, weighing only 249 g, had no measurable impact on bat activity when conducting acoustic surveys^22^. Nonetheless, that study did not directly test the use of acoustics on miniature drones due to their small payload capacity, making it challenging to suspend a recording device from the drone.

Here, we investigate how habitat, drone altitude, and drone movement influence bat sampling efficiency with a miniature drone for *M. septentrionalis*, *M. lucifugus*, and *M. leibii* (MYSP complex), *E. fuscus* and *L. noctivagans* (EPNO complex), and all bats. First, we predicted that if *E. fuscus* prefers the presence of dwellings^23,24^, and we acoustically sample bats in a variety of habitats, then more EPNO bats will be detected in habitats containing human-made structures, such as houses and sheds. Second, we predicted that if *E. fuscus* and the MYSP complex exhibit differences in body size and flight morphology^25–27^, and we acoustically sample bats using a drone at varying altitudes from 5 to 63 m, then the EPNO and MYSP complex will differentially occupy this altitude range of the air column. Third, we predicted that if stationary handheld recording devices detect more bats than mobile recording devices^28,29^ and we sample bats using a hovering (stationary) drone and a moving drone, then bats will be detected more with the former. Overall, our goal is to develop a non-invasive, efficient and effective technique for acoustically sampling bats using a miniature drone.

## Results

### All bats

**Fig. 1:**
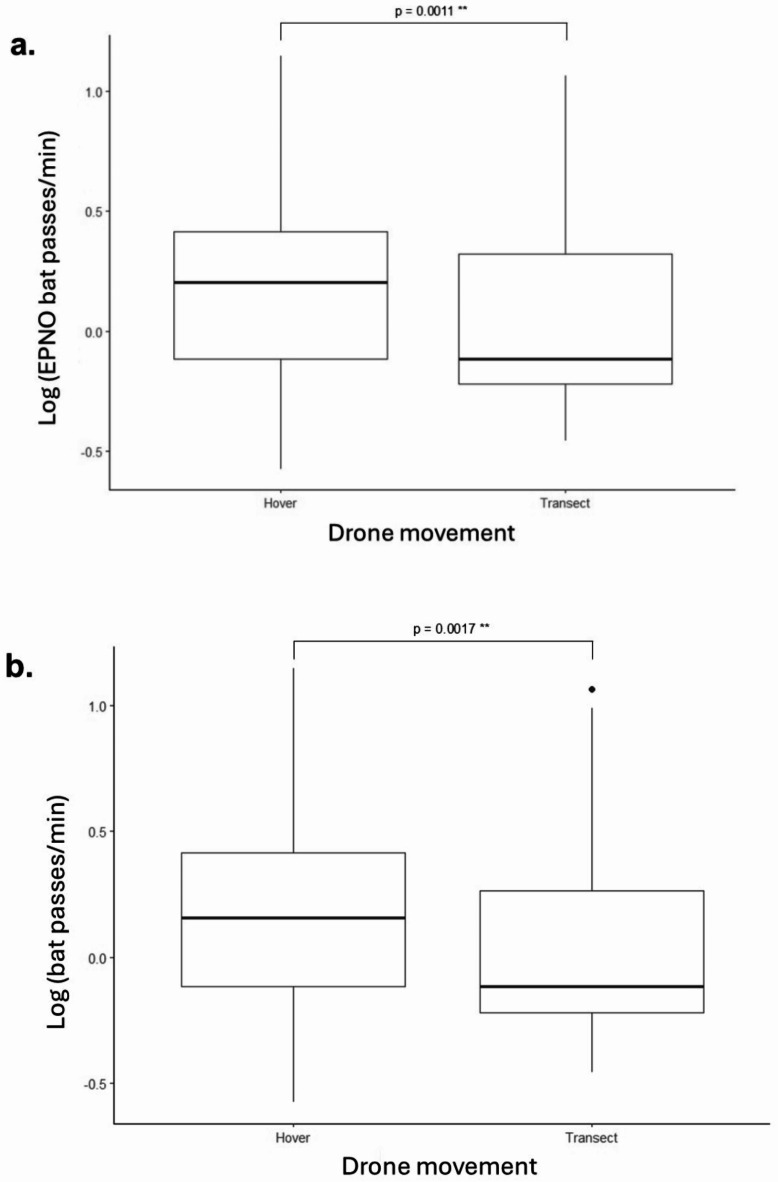
Log-transformed counts of bat passes per minute compared between a hovering drone and a moving drone for *E. fuscus* and *L. noctivagans* (**A**) and all bats (**B**). Only trials where at least one *E. fuscus* or *L. noctivagans* was detected with a drone are included in A and trials where at least one bat of any species was detected with a drone are included in B. Statistical significance is marked by ** (*p* < 0.01). A hovering drone detected significantly more bat passes per minute than a moving drone for both species groups.

A total of 1220 recordings were manually analyzed, including 392 recordings of bats during drone trials (*n* = 392 for all bats). Drone detections of *Lasiurus borealis* (*n* = 2), *L. cinereus* (*n* = 2), and *Perimyotis subflavus* (*n* = 1) were included in the analysis of all bats but were not analyzed as separate species groups due to their low sample sizes. Bat passes per minute when bats were present, but not bat presence, increased with a hovering drone (see Supplementary Table S1 online). Specifically, a hovering (stationary) drone detected more bats per minute than a moving drone for all bats (t_57.02_ = -3.30, *p* = 0.002) (see Fig. 1). There was no significant difference in total bat presence between sampling with a hovering drone and a moving drone (z = -0.861, *p* = 0.39). Bats were significantly more likely to be present in building sites compared to coniferous forests (Tukey’s test, *p* = 0.021), and building sites compared to wetlands (Tukey’s test, *p* = 0.028). There was no significant difference in the number of bat passes per minute between the different habitat types (see Supplementary Table S2 online). Drone altitude did not significantly impact bat presence (z = 0.17, *p* = 0.86) or the number of bat passes per minute (t_69.56_ = 1.16, *p* = 0.25) for all bats.

### Big brown bat/silver-haired bat (EPNO) complex

EPNO bats (*E. fuscus* and *L. noctivagans*) were detected 360 times using a drone (*n* = 360). Bat passes per minute when a bat was present, but not bat presence, was impacted by drone movement (see Supplementary Table S1 online). Specifically, a hovering (stationary) drone detected more bats from the EPNO complex than a moving drone (t_46_ = -3.49, *p* = 0.001) (see Fig. 1). There was no significant difference in bat presence between sampling with a hovering drone and a moving drone for the EPNO complex (z = -0.92, *p* = 0.36). Bats in the EPNO complex were significantly more likely to be present in building sites compared to coniferous forests (Tukey’s test, *p* = 0.048), building sites compared to wetlands (Tukey’s test, *p* = 0.009), and deciduous forests compared to wetlands (Tukey’s test, *p* = 0.024). There was no significant difference in the number of EPNO bat passes per minute between the different habitat types (see Supplementary Table S3 online). Drone altitude did not significantly impact bat presence (z = 1.105, *p* = 0.27) or the number of bat passes per minute (t_57_ = 1.02, *p* = 0.31).

### Myotis (MYSP) complex

**Fig. 2:**
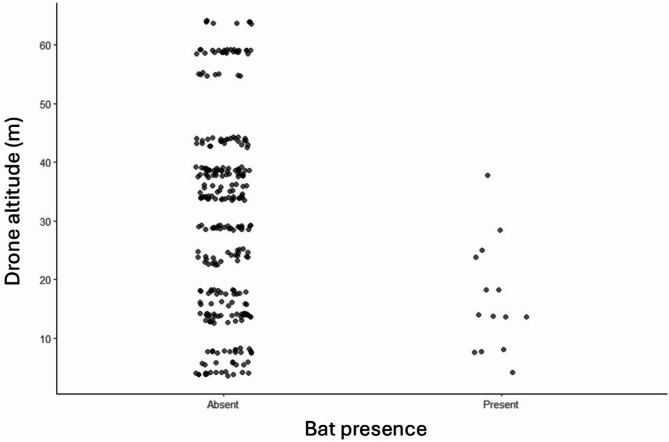
Presence and absence of the MYSP complex at varying altitudes detected with a drone. Each point represents one drone sampling trial. Statistical significance is marked by ** (p < 0.01). The MYSP complex includes* M. septentrionalis*,* M. lucifugus*, and* M. leibii*. The drone detected significantly more MYSP bats at lower altitudes.

MYSP bats (*M. septentrionalis*, *M. lucifugus*, and *M. leibii*) were detected 27 times using a drone (*n* = 27). Bat presence, but not the number of bat passes per minute when bats were present, decreased with altitude (see Supplementary Table S1 online). Specifically, the presence of MYSP bats was significantly more likely to be detected with a drone flying at a lower altitude (z = -2.60, *p* = 0.01) (see Fig. 2). There was no significant impact of drone altitude on the number of MYSP bat passes per minute (t_13.57_ = -1.54, *p* = 0.15). There was no significant difference in bat presence (z = 0.014, *p* = 0.99) or the number of bat passes per minute (t_13.70_ = 0.62, *p* = 0.54) for the MYSP complex between sampling with a hovering drone and a moving drone. There was no significant difference in bat presence or the number of bat passes per minute between the different habitat types for the MYSP complex (see Supplementary Table S4 online).

## Discussion

Drone altitude impacted the presence of *Myotis* bat detections, with the presence of the MYSP complex only detected at altitudes less than 40 m. This finding is consistent with our prediction that the bat complexes would differentially occupy sectors of the air column. However, contrary to our prediction, there was no effect of altitude on the presence or number of bat detections for the EPNO complex or all bats. This could be because, although the EPNO complex was present at high altitudes over the forest canopy, they were also frequently found near buildings, and therefore at lower altitudes.

Drone movement impacted the number of bat detections, with a hovering drone detecting more bats per minute than a moving drone for the EPNO complex and all bats. This finding aligns with our prediction and previous studies that found that stationary bat recording devices are more efficient than mobile devices^28,29^. Our results confirm that this is also the case when recording bats with drones, and we encourage future drone-based bat studies to use hovering point counts when conducting drone-based bat surveys.

Habitat type impacted the presence and number of bat detections, with both the EPNO complex and all bats more likely to be present near man-made structures than in coniferous forests or wetlands. This aligns with our prediction and previous findings that *E. fuscus* is strongly associated with urban environments^23,24^. However, this is the first study to confirm this habitat preference with drone-based acoustic surveys. Despite the study having taken place at a nature reserve with very little human disturbance, bats were still more likely to be found near the few man-made structures, such as houses and sheds. This reiterates the ability of *E. fuscus* to adapt to urban environments and outcompete other bat species in such habitats^30^ and emphasizes the importance of conserving natural spaces for other bat species.

This study is the first to confirm that miniature drones are an effective tool for sampling bats. Finding a new method for monitoring cryptic endangered species is crucial for future research and conservation efforts. This method, which uses a miniature drone model that was found to have no significant effect on bat activity^22^, is relatively inexpensive and non-invasive, using equipment that can easily be purchased by other researchers or citizen scientists. It is also a relatively safe method of accessing high altitudes and remote areas at night. Sampling bats with miniature drones will become more feasible as drones become quieter, further minimizing the impact on wildlife. As drone technology advances, miniature drones will have a greater payload capacity capable of supporting heavier, higher-performing recording devices, and will have longer battery lives, allowing for longer sampling sessions. We encourage future studies and monitoring projects to employ this method of acoustic sampling using miniature drones. Future research could focus on using miniature drones to sample bats at higher altitudes (over 60 m) to better understand how bats occupy the airspace above the forest canopy.

## Methods

We conducted the study on the property of Kenauk, a nature reserve in Montebello, Quebec, Canada. All experiments were approved by the McGill University Animal Care Committee as Animal Use Protocol MCGL-2021-8205. Previous research at Kenauk has detected all eight species of native Quebec bats on the property^6^. The sampling period ranged from July 12 to August 23, 2023. We sampled bats between 21:00 and 00:00 and did not sample if it was raining or if there were winds above 15 km/h. We sampled bats at 12 different locations within Kenauk: three wetland sites, three building sites (a marina, a garage, and a house), three coniferous forest sites, and three deciduous forest sites. We sampled each site four or five times. A random number generator randomized the order of sites for each night. We used an iPod Touch 7 with the Wildlife Acoustics app including integrated Kaleidoscope Pro Analysis Software and an Echo Meter Touch 2 as the recording device.

### Drone sampling

**Fig. 3:**
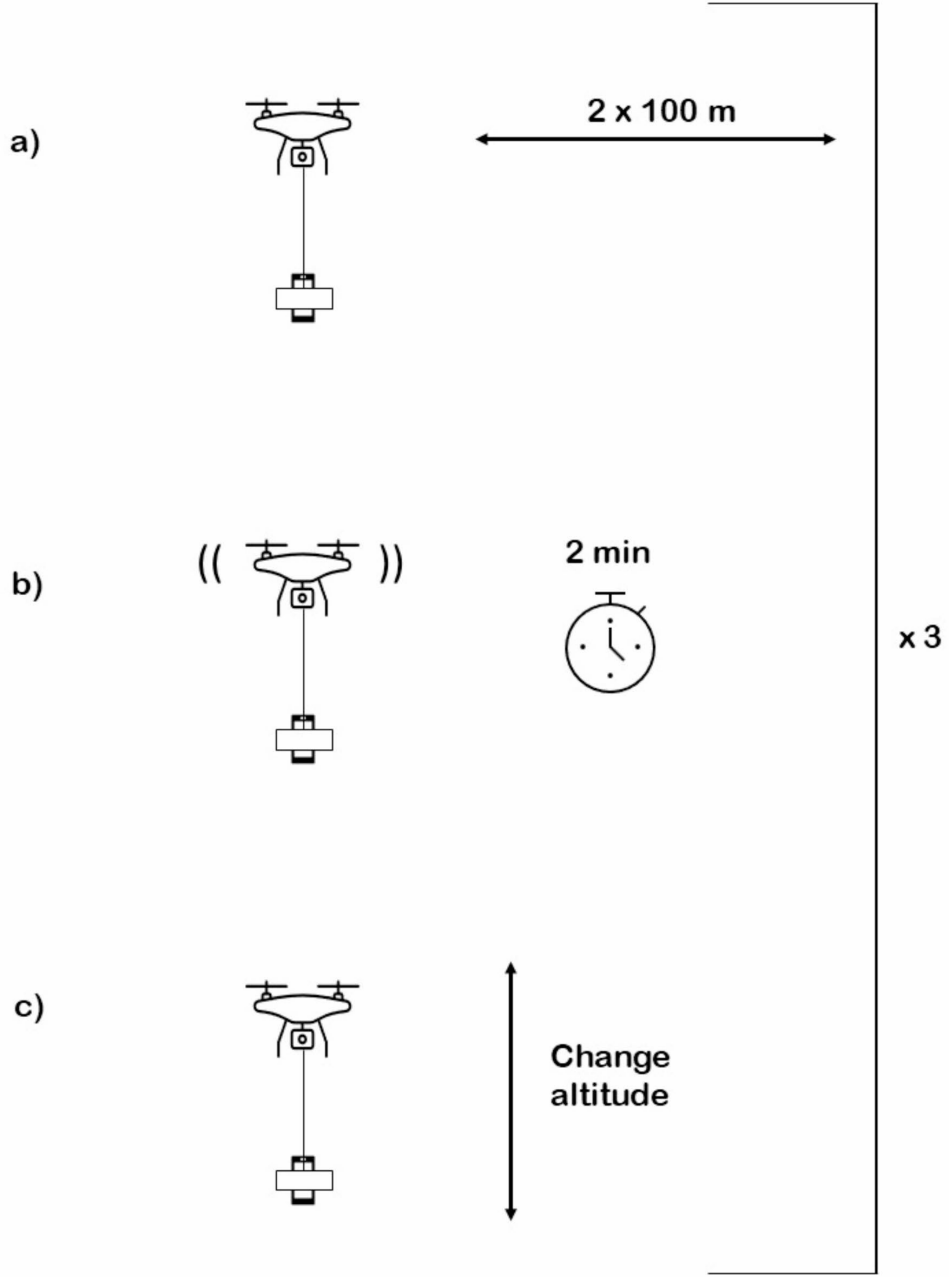
Acoustic sampling procedure. The drone method consisted of flying a 100 m transect six times. Every 200 m, once the drone returned to its starting point (**a**), it would hover for two minutes (**b**). After hovering, the drone would move vertically to the next altitude (**c**) and repeat the same process at the new altitude, for a total of three altitudes per session

The pilot possessed a Basic Operations Small Remotely Piloted Aircraft System Pilot Certificate issued by Transport Canada. Before initiating the drone mission (see Autonomous Flight Programming & Piloting section), a field technician started recording on the Wildlife Acoustics app and inserted the recording device into the foam package (see Drone Setup section). The drone trajectory consisted of a 100 m transect repeated six times (see Fig. 3). Each drone mission included three altitudes: the lowest safe altitude, the lowest altitude plus 10 m, and the lowest altitude plus 30 m. We determined the lowest safe altitude during the day by flying the drone over the tallest obstacle at that site, slowly lowering it to the lowest safe height, and noting the altitude indicated on the drone controller. The altitudes ranged from 5 to 63 m. We randomly assigned and programmed the order of the altitudes flown before each sampling session (see Autonomous Flight Programming & Piloting section). We took note of the time at which the drone started the route, began a new transect or hover, and ended the route to later categorize the recordings. When the drone landed, we stopped recording and changed the drone batteries for the next site.

### Drone setup

**Fig. 4:**
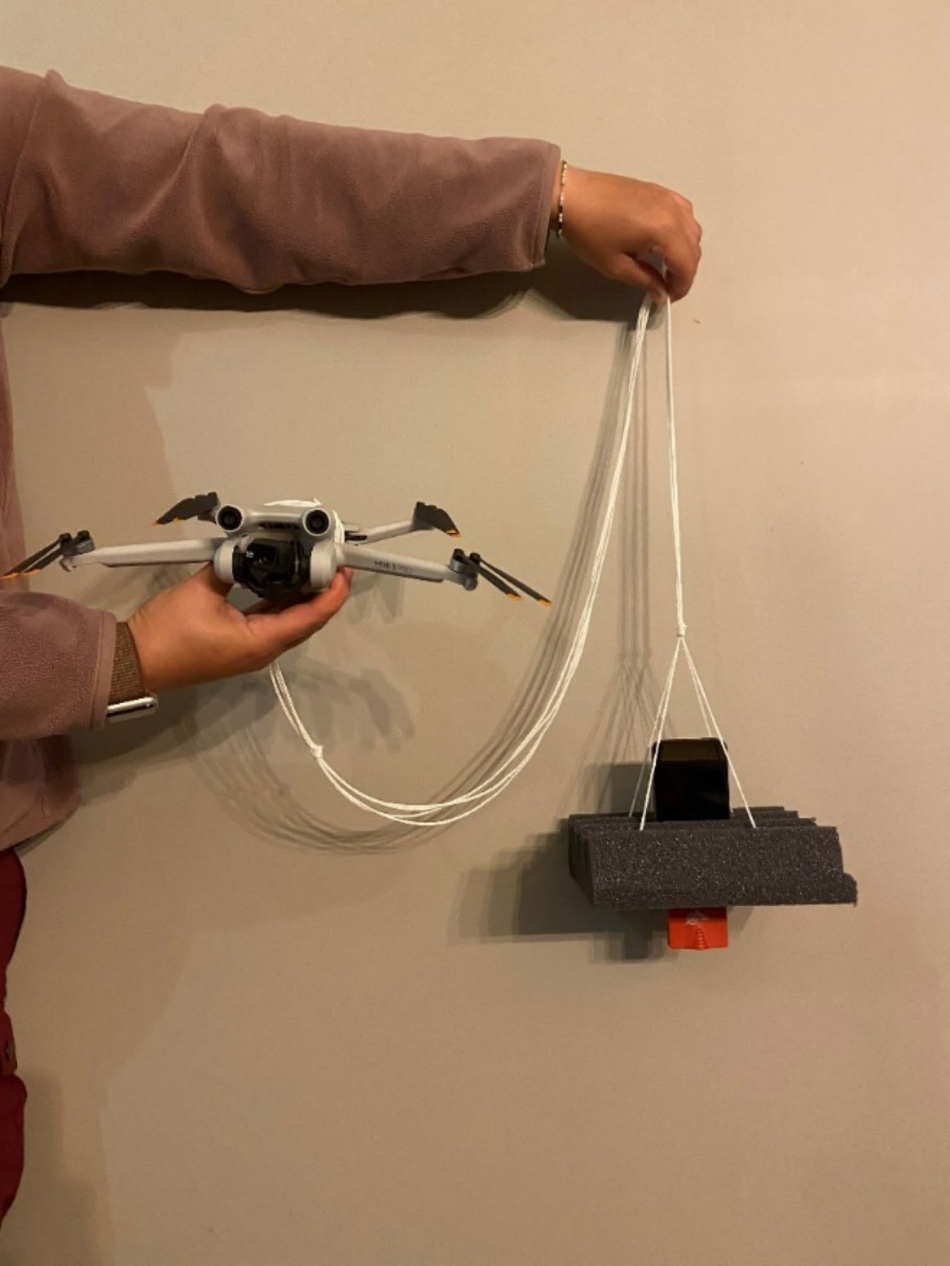
Drone setup. We placed an iPod Touch 7 and Echo Meter Touch 2 into a 16 by 18 cm cutout of Auralex 2-inch Studiofoam Wedge acoustic foam. We glued a 10 cm by 10 cm piece of cardboard to the middle of the foam square to reinforce the area supporting the iPod. We suspended the acoustic foam package 135 cm from the drone with nylon string. We secured one end of the string to the four corners of the cardboard and tied the other end around the middle of the drone. We taped the string to the drone to prevent loose ends from getting caught in the propellers. We avoided covering the bottom sensor of the drone.

We employed the DJI Mavic Mini 3 Pro due to its lightweight design (249 g) and high payload capacity. This model choice aligns with the findings of Kuhlmann et al.^22^, which demonstrated no significant impact on bat activity when using the DJI Mavic Mini 2, a similar model of drone the same size as the DJI Mavic Mini 3 Pro. Throughout our preliminary tests, the DJI Mavic Mini 3 Pro was the only miniature drone capable of supporting the weight of the recording device. In contrast, the payload dragged down the DJI Mavic Mini 2 and DJI Mavic Mini 3. The DJI Mini 3 Pro Intelligent Flight Battery Plus was required for the drone to complete the mission in full without changing the battery. To limit detected drone noise, we surrounded the recording device with acoustic foam^6^ and distanced the recording device from the drone^9,20^ by 135 cm, a distance far enough to separate the recording device from the drone, but short enough to prevent the package from swinging (see Fig. 4).

### Autonomous flight programming & piloting

We mapped out an autonomous drone route for each site using DroneLink software to reduce nocturnal manual piloting and ensure route consistency. We designed and tested the autonomous routes during the daytime to ensure that the drone would avoid obstacles. We randomized and preprogrammed the order of altitudes flown on DroneLink before each sampling night. We downloaded the autonomous routes to the DJI RC Pro controller before heading out to the field to initiate the autonomous flights at the remote sites. The pilot could end the autonomous route and take control of the drone at any time. When the autonomous route was completed, the pilot manually took control of the drone to land it. Landing usually required the pilot to gently direct the drone from side to side to swing the parcel away from the bottom sensor of the drone and lower it to the ground.

### Bat identification

The Wildlife Acoustics app automatically identified the bat recordings using Kaleidoscope Pro Analysis Software. However, automatic identification software is not sufficiently accurate to be solely relied upon for bat species identification^31–33^. Therefore, we manually identified all the recordings using cluster analysis and the spectrogram viewer on Kaleidsocope version 5.6.4. We identified the species or complex based on the frequency, shape, and duration of the call using the guides from Charbonneau et al.^33^ and Szewczak et al.^31^ (see Supplementary Fig. S1 online). To avoid identification errors, we grouped species with similar call parameters into two complexes based on spectrogram similarities, as seen in^6,22,23^. The EPNO complex included *E. fuscus* and *L. noctivagans* and the MYSP complex included *M. lucifugus*, *M. septentrionalis*, and *M. leibii.* The spectrograms of the species within each group are known to be highly similar, making differentiation challenging^34^.

### Statistical analysis

We used R version 4.3.1 for the statistical analyses^35^. We fit linear models using the lme4 package^36^. We fit each model separately for the *Myotis* complex, the EPNO complex, and all species. We used AIC values to identify the best-fitting model for each group. When habitat type was included in the best-fitting model, we used a Tukey HSD post hoc test with the glth function from the multcomp package^37^ to test the difference in bat presence or the number of bat passes per minute between each habitat type.

To test whether habitat type, recording device altitude, and drone movement affected bat presence, we used binomial generalized linear mixed models with habitat, altitude, and movement as fixed effects and sampling trial as a random effect with the response variable as bat presence (1) or absence (0). To test whether habitat type, recording device altitude, and drone movement affected bat passes per minute, we used linear mixed models with habitat, altitude, and movement as fixed effects and trial as a random effect with the response variable as the number of bat passes per minute. Only trials where at least one bat of the complex of interest was detected were included in the models for bat passes per minute.

## Electronic supplementary material

Below is the link to the electronic supplementary material.

Supplementary Material 1

Supplementary Material 2

## Data Availability

All data generated or analysed during this study will be included in this published article (and its “Supplementary information” files).
