# Supplementary material for: Acoustic monitoring with miniature drones shows reduced Myotis bat occurrence with altitude and drone movement
Source: Sci Rep. 2025 Apr 10;15:12307. doi: 10.1038/s41598-025-96255-5 (PMC11985985; doi:10.1038/s41598-025-96255-5)
Supplement: Supplementary file 1 — Supplementary Material 1 [file 41598_2025_96255_MOESM1_ESM.docx]

**Supplementary Information**

**Supplementary Table S1.** Akaike information criterion (AIC) for bat presence and bat passes per minute models. All models for bat passes per minute are linear mixed models with the response variable of the log-transformed number of bat passes per minute. Models for presence are generalized linear mixed models, with the binomial response variable of 0 (no bats) or 1 (bats present). The sampling trial is included as a random effect in all the models. Bolded AIC values are the best-fitting models for each category. EPNO is the complex including *E. fuscus* and *L. noctivagans*. MYSP is the *Myotis* complex including *M. septentrionalis*, *M. lucifugus*, and *M. leibii*. All bats includes all of the above species, as well as *L. borealis* and *L. cinereus*.

| Model Parameters | MYSP | EPNO | All Bats | MYSE | EPNO | All Bats |
| --- | --- | --- | --- | --- | --- | --- |
|  | Bat Presence with Drone | | | Bat Passes/min with Drone | | |
| Habitat, alt, movement | 109.4005 | 264.3212 | 295.5004 | 33.4693 | 43.7284 | 47.5707 |
| Habitat, altitude | 107.3062 | 263.1236 | 294.1478 | 22.3178 | 52.4383 | 55.3839 |
| Habitat, movement | 113.7991 | 263.5025 | 293.4046 | 21.8402 | 41.8424 | 45.3448 |
| Altitude, movement | 105.8914 | 272.8292 | 301.7304 | 9.9446 | 39.0427 | 45.8392 |
| Habitat | 111.7168 | **262.2788** | **292.0676** | 14.1778 | 50.2186 | 53.1306 |
| Altitude | **103.8379** | 271.7063 | 300.4577 | 5.5507 | 47.7826 | 53.4286 |
| Movement | 300.4577 | 273.0170 | 299.7089 | 7.1978 | **38.4139** | **45.1179** |
| Null | 110.6189 | 271.8592 | 298.4460 | **3.5326** | 46.5409 | 52.5316 |

**Supplementary Table S2**. Tukey HSD post hoc test results comparing bat passes per minute and bat presence between different habitat types for all bat species. Only trials where at least one bat of any species was detected were included for the bat passes per minute. The bat species include *E. fuscus*, *L. noctivagans*, *M. septentrionalis*, *M. lucifugus*, *M. leibii*, *L. borealis,* and *L. cinereus*. Statistical significance is marked by * (p < 0.05). Bats were significantly more likely to be present in building sites compared to coniferous forests and building sites compared to wetlands. There was no significant difference in the number of bat passes per minute between the different habitat types.

| Difference of Levels | Estimate | SE | Z-Value | P-Value |
| --- | --- | --- | --- | --- |
|  | Bat Passes/min with Drone | | | |
| Coniferous - Building | -0.0859 | 0.1572 | -0.547 | 0.947 |
| Deciduous - Building | 0.3096 | 0.1337 | 2.316 | 0.092 |
| Wetland - Building | 0.0164 | 0.1611 | 0.102 | 0.999 |
| Deciduous - Coniferous | 0.3955 | 0.1699 | 2.327 | 0.090 |
| Wetland - Coniferous | 0.1023 | 0.1923 | 0.532 | 0.950 |
| Wetland - Deciduous | -0.2932 | 0.1737 | -1.688 | 0.325 |
|  | Bat Presence with Drone | | | |
| **Coniferous - Building** | **-2.2769** | **0.7916** | **-2.876** | **0.021 *** |
| Deciduous - Building | -0.5991 | 0.7230 | -0.829 | 0.840 |
| **Wetland - Building** | **-2.2165** | **0.7967** | **-2.782** | **0.028 *** |
| Deciduous - Coniferous | 1.6778 | 0.8276 | 2.027 | 0.177 |
| Wetland - Coniferous | 0.0604 | 0.8678 | 0.070 | 0.999 |
| Wetland - Deciduous | -1.6173 | 0.8303 | -1.948 | 0.207 |

**Supplementary Table S3**. Tukey HSD post hoc test results comparing EPNO bat passes per minute and the presence of EPNO bats between different habitat types. Only trials where at least one EPNO bat was detected were included for the bat passes per minute. The EPNO complex includes *E. fuscus* and *L. noctivagans.* Statistical significance is marked by * (p < 0.05) and ** (p < 0.01). Bats in the EPNO complex were significantly more likely to be present in building sites compared to coniferous forests, building sites compared to wetlands, and deciduous forests compared to wetlands. There was no significant difference in the number of EPNO bat passes per minute between the different habitat types.

| Difference of Levels | Estimate | SE | Z-Value | P-Value |
| --- | --- | --- | --- | --- |
|  | Bat Passes/min with Drone | | | |
| Coniferous - Building | -0.0764 | 0.1784 | -0.428 | 0.973 |
| Deciduous - Building | 0.2425 | 0.1453 | 1.670 | 0.331 |
| Wetland - Building | 0.0999 | 0.2195 | 0.455 | 0.967 |
| Deciduous - Coniferous | 0.3189 | 0.1900 | 1.679 | 0.326 |
| Wetland - Coniferous | 0.17640 | 0.2514 | 0.702 | 0.893 |
| Wetland - Deciduous | -0.1426 | 0.2291 | -0.622 | 0.922 |
|  | Bat Presence with Drone | | | |
| **Coniferous - Building** | **-2.0948** | **0.8123** | **-2.579** | **0.048 *** |
| Deciduous - Building | -0.2002 | 0.7229 | -0.277 | 0.992 |
| **Wetland - Building** | **-2.8461** | **0.9064** | **-3.140** | **0.009 **** |
| Deciduous - Coniferous | 1.8946 | 0.8481 | 2.234 | 0.113 |
| Wetland - Coniferous | -0.7513 | 0.9721 | -0.773 | 0.865 |
| **Wetland - Deciduous** | **-2.6458** | **0.9365** | **-2.825** | **0.024*** |

**Supplementary Table S4.** Tukey HSD post hoc test results comparing MYSP bat passes per minute and the presence of MYSP bats between different habitat types. Only trials where at least one MYSP bat was detected were included for the bat passes per minute. The MYSP complex includes *M. septentrionalis*, *M. lucifugus*, and *M. leibii*. There is no significant difference in MYSP bat passes per minute or presence between habitat types.

| Difference of Levels | Estimate | SE | Z-Value | P-Value |
| --- | --- | --- | --- | --- |
|  | Bat Passes/min with Drone | | | |
| Coniferous - Building | -7.669e-02 | 0.1807 | -0.424 | 0.972 |
| Deciduous - Building | -7.669e-02 | 0.1807 | -0.424 | 0.972 |
| Wetland - Building | 2.303e-01 | 0.1043 | 2.207 | 0.113 |
| Deciduous - Coniferous | 2.776e-17 | 0.2410 | 0.000 | 1.000 |
| Wetland - Coniferous | 3.070e-01 | 0.1905 | 1.612 | 0.355 |
| Wetland - Deciduous | 3.070e-01 | 0.1905 | 1.612 | 0.355 |
|  | Bat Presence with Drone | | | |
| Coniferous - Building | -2.1882 | 1.2921 | -1.694 | 0.321 |
| Deciduous - Building | -2.1055 | 1.2761 | -1.650 | 0.344 |
| Wetland - Building | -0.8851 | 0.9908 | -0.893 | 0.804 |
| Deciduous - Coniferous | 0.0828 | 1.5924 | 0.052 | 1.000 |
| Wetland - Coniferous | 1.3032 | 1.3603 | 0.958 | 0.769 |
| Wetland - Deciduous | 1.2204 | 1.3509 | 0.903 | 0.799 |

**
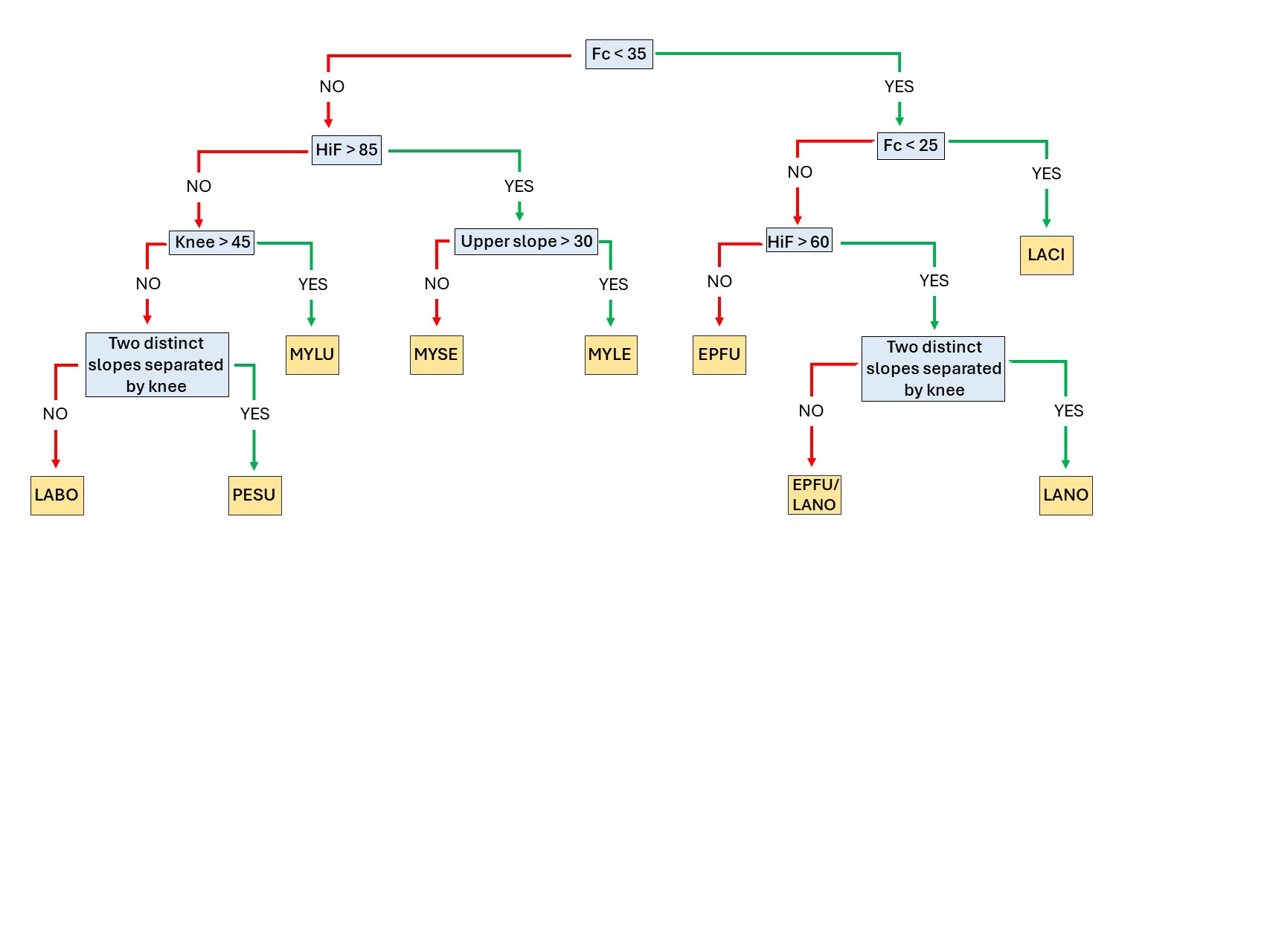
**

**Supplementary Figure S1**. Decision tree for manually vetting Quebec bat calls. All values are in kHz. Fc is the frequency of the call at its lowest slope, HiF is the highest frequency of the call, the knee is a pronounced inflection in the slope of the call, and the upper slope is the slope of the call between HiF and the knee. The yellow boxes each represent a species: LABO is *L. borealis*, PESU is *P. subflavus*, MYLU is *M. lucifugus*, MYSE is *M. septentrionalis*, MYLE is *M. leibii*, EPFU is *E. fuscus*, LANO is *L. noctivagans*, and LACI is *L. cinereus*. Values adapted from Szewczak, J. M., Corcoran, A., Kennedy, J. P., Ormsbee, P. C., & Weller, T. J. (2011). Echolocation Call Characteristics of Eastern US Bats. <https://www.sonobat.com/download/EasternUS_Acoustic_Table_Mar2011.pdf>
